# Prevalence and risk factors of domestic violence and its impacts on women’s mental health in Gilgit-Baltistan, Pakistan

**DOI:** 10.12669/pjms.36.4.1530

**Published:** 2020

**Authors:** Hina Hussain, Sadiq Hussain, Samar Zahra, Talib Hussain

**Affiliations:** 1 Mrs. Hina Hussain, M.Phil. Department of Behavioral Sciences, Karakoram International University, Gilgit, Pakistan; 2 Dr. Sadiq Hussain, PhD. Department of Behavioral Sciences, Karakoram International University, Gilgit, Pakistan; 3 Mrs. Samar Zahra, M.Phil. Department of Behavioral Sciences, Karakoram International University, Gilgit, Pakistan; 4 Dr. Talib Hussain, PhD. Department of Business Management, University of Baltistan, Skardu, Pakistan

**Keywords:** Domestic violence, Gilgit-Baltistan, Mental health, Prevalence, Women

## Abstract

**Objective:**

To assess the prevalence of domestic violence, associated risk factors, and its impacts on women’s mental health in Gilgit-Baltistan (GB), Pakistan.

**Methods:**

This is a sequential explanatory strategy that is a mixed-method research design was conducted at Department of Behavioral Sciences, Karakoram International University Gilgit from January 2017 to June 2018 on 160 married women. Quantitative data were collected using Karachi domestic violence screening scale and mental health inventory and qualitative data were collected through interview guides. Descriptive and inferential statistical techniques were applied to analyze quantitative data while qualitative data were analyzed using thematic analysis.

**Results:**

Married women in GB reported higher levels of domestic violence (88.8%; psychological (69.4%), physical (37.5%) & sexual (21.2%). Abused women reported lower levels of mental health (*t*=3.19, *p*=0.00); psychological wellbeing (*t*=2.03, *p*=0.04), general positive affect (*t*=2.09, *p*=0.03), and life satisfaction (*t*=2.39, *p*=0.01) and higher levels of psychological distress (*t*=3.27, *p*=0.00), anxiety (*t*=3.06, *p*=0.00), depression (*t*=2.60, *p*=0.01), and loss of emotional/behavioral control (*t*=3.05, *p*=0.00) as compared to non-abused women. Risk factors behind domestic violence were identified as; poverty, the influence of in-laws, second marriage, stepchildren, forceful intimate relationships, husband’s irresponsibility, and addiction, and handicapped children.

**Conclusions:**

We found higher level of domestic violence, associated risk factors, and poor mental health of abused women in GB.

## INTRODUCTION

Domestic violence as the supremacy exploited by one adult in a relationship to control another is not simply an argument, but the abuser uses physical and sexual violence, emotional offences, and economic denial against women.^1^ It is a global issue, which is prevalent throughout nations, socio-economic classes, cultures, and race.^1^ According to the World Health Organization report, 35% women globally have experienced either physical and/or sexual violence. Globally, around one third of all women experienced either physical or sexual violence, and in some regions, the figure increased to 38%.^2^ The worldwide prevalence of non-partner sexual violence only in 2010 was 7.2%. The highest prevalence was in African countries and lowest in Asian countries while limited data were available regarding Europe, Middle East, Asia pacific, and high-income countries.^3^

In Pakistan, domestic violence appeared in different forms, which, ranged from shouting to the use of weapons, including nonconsensual sex and only 3.2% women did not report any type of domestic violence.^4^ In rural Pakistan, the prevalence of physical abuse against women was 56% while in urban settings, the lifetime prevalence of physical, sexual, and psychological abuse were 57.6%, 54.5%, and 83.6% respectively.^5^,^6^ Another study that reviewed empirical studies published from 1998 to 2008 found that 30% to 79% of women reported intimate partner violence in Pakistan.^7^

Distressing impacts of domestic violence are limited not only to physical injuries but contribute to the ill health of women and weak psychological and emotional wellbeing.^1^ Victims of intimate partner violence reported the increasingly adverse effects on their mental health; depression, posttraumatic stress disorder, and anxiety as compared to those who never experienced intimate partner violence.^8^ Findings of a meta-analysis revealed that the weighted mean prevalence of mental health issues among abused women were; 47.6%, 17.9%, 63.8%, 18.5%, and 8.9% for depression, suicidality, PTSD, alcohol abuse, and drug abuse respectively.^9^ Other researchers claimed that domestic violence is linked with many mental disorders such as; anxiety, depression, PTSD, eating disorders, and even psychosis.^10^ In rural Pakistan, 98% of women reported mental tension due to the maltreatment by their husbands.^5^

Keeping in view, the unrelenting negative consequences of domestic violence on women’s mental health, researchers have identified a number of risk factors such as cultural and social norms, religious practices, and economic and political circumstances.^1^ Specific to the Pakistani context, the identified risk factors associated with domestic violence against women were; women’s low education and low empowerment, miss concepts of Islamic reflections and customary norms such as justifying honor killing, and poverty and the prevalent custom of the undue-traditional dowry system in the society.^7^ Another study reported in-laws, disobedience and arguments with husband, husbands’ addiction, extra marital relationship and infertility as risk factors of domestic violence.^11^ In rural Pakistan, risk factors of domestic violence against women were; age, education, and income of women while in urban Pakistan different risk factors for different types of violence were classified, i.e. husband’s low education, unskilled work, and five or more family members in the family for physical violence, wife’s low education, low socioeconomic status, and five or more family members in the family for sexual violence, and husband’s unskilled working status and low socioeconomic status of the family for psychological violence.^5^,^6^

The reviewed literature shows that the prevalence, causes, and the consequences of domestic violence against women are well-studied globally as well as within Pakistan, but an actual condition in Gilgit-Baltistan (GB) has not been studied. GB, the region of Pakistan, with difficult access, being far-flung, and lacking basic necessities of life remained unexplored in terms of the prevalence of domestic violence, its risk factors, and impacts on women’s mental health. Therefore, this first scientific study was conducted with objectives to assess the prevalence, risk factors, and impact of domestic violence on women’s mental health in GB.

## METHODS

This is a sequential explanatory strategy that is a mixed-method research design was conducted at Department of Behavioral Sciences, Karakoram International University Gilgit from January 2017 to June 2018. In the first phase of study, after approval of research protocols from institutional ethical review committee (KIU-IMARC/2019/252 dated July 19, 2019), quantitative data were collected from 160 married women age ranged from 18-50 years. Karachi domestic violence screening Scale-Urdu and mental health inventory were used to assess domestic violence and mental health respectively.^12^,^13^ In the second phase, 142 (88.8%) women were included who were characterized/screened as abused based on the result of the first phase. A self-constructed interview guide was used to conduct in-depth interviews (IDIs) and focus group discussions (FGDs) to identify risk factors of domestic violence. Demographic information form was used to collect participants’ demographic information like age, education, family system, married years, occupation, and monthly income of respondents.

In the first phase, collected data were analyzed using descriptive statistical techniques. In this phase, respondents were classified into two groups; abused and non-abused women based on their scores on KDVSS-U. Then using independent sample *t-test* compared both groups’ mental health by using Statistical Package for Social Sciences (SPSS, v-20). In the second phase, qualitative data were analyzed using content analysis to identify the underlying risk factors of domestic violence.^14^ Level of significance *p*≤0.05 was considered significant and effect sizes of associations were also calculated “*d*”.

## RESULTS

Out of the total participants, 120(75.0%) belong to age rang 18-33 years, 73(46.8%) have reported more than 10 married years, 75(49.9%) have obtained graduation and above education level, 85(53.1%) were housewives, 82(51.3%) were from joint family background, and a good number of them 72(45.0%) reported less than ten thousand monthly incomes (Table-I).

**Table-I:** Demographic characteristic of participant.

Variables	Ranges	n (%)
Age in years	18-33	120(75.0%)
	34-50	40(25.0%)
Married years	1-5	60(37.5%)
	6-10	27(16.9%)
	Above 10	73(45.6%)
Level of education	Illiterate	19(11.9%)
	Literate	9(5.6%)
	High school education	57(35.6%)
	Graduation & above	75(46.8%)
Occupation	Housewives	85(53.1%)
	Teaching	37(23.1%)
	Non-skilled work	38(23.8%)
Family system	Nuclear	78(48.8%)
	Joint	82(51.3%)
Family income in PKRS	<10000	72(45.0%)
	11000-20000	51(32.0%)
	21000-30000	29(18.0%)
	>30000	8(5.0%)

According to overall KDVSS scale scores 142(88.8%) women were found abused. Physical abuse, psychological abuse, and sexual abuse were found among 60(37.5%), 111(69.4%) and 34 (21.2%) women respectively (Table-II).

**Table-II:** Abused and non-abused women on KDVSS.

Variables	Range	n (%)	Interpretation
KDVSS	0-2	18 (11.2%)	Non-abused
	≥3	142 (88.8%)	Abused
Physical abuse	0-2	100 (62.5%)	Non-abused
	≥3	60 (37.5%)	Abused
Psychological abuse	0-2	49 (30.6%)	Non-abused
	≥3	111 (69.4%)	Abused
Sexual abuse	0-2	126 (78.8%)	Non-abused
	≥3	34 (21.2%)	Abused

Abused women reported lower levels of mental health, psychological wellbeing, general positive affect, and life satisfaction and higher levels of psychological distress, anxiety, depression, and loss of emotional/ behavioral control as compared to non-abused women. The effect size was medium for mental health and psychological distress and small for psychological wellbeing, general positive affect, life satisfaction, anxiety, depression, and loss of emotional/behavioral control (Table-III).

**Table-III:** Comparison of mental health between abused and non-abused women.

Variables	Non-abused women		Abused women				

	M	SD	M	SD	t	p	d
Mental health	177.4	26.36	154.5	28.93	3.19	0.00	0.51
Psychological wellbeing	68.3	11.38	62.22	11.97	2.03	0.04	0.32
Psychological distress	50.4	21.01	69.08	23.02	3.27	0.00	0.52
Anxiety	21.39	10.03	28.58	9.29	3.06	0.00	0.49
Depression	8.17	4.16	11.46	5.16	2.60	0.01	0.41
Loss of emotional behavioral control	17.22	6.98	23.35	8.13	3.05	0.00	0.49
General positive affect	50.50	9.39	45.32	9.93	2.09	0.03	0.33
Emotional ties	6.56	2.53	6.15	2.46	0.66	0.51	0.11
Life satisfaction	5.61	1.24	4.75	1.47	2.39	0.01	0.38

Based on qualitative data, seven major themes have been generated while exploring the risk factors of domestic violence. Among the most frequent responses, poverty comes out to be the major bone of contention (89%). Second, most frequently reported category (67%) was the influence of in-laws in exacerbating disagreements among spouses. The second marriage was another major risk factor of quarrel among couples (50%); either husband went for second marriage, or the wife herself was the second wife and step children creates issues. Therefore, 42% reported stepchildren as a reason for the quarrel. The forceful intimate relationship (25%) along with irresponsibility of husband (15%) and addicted husband (15%), handicapped children (7%), husband’s demand of financial assistance from wife’s family (6%), and husband reluctance for children (6%) were also found risk factors of domestic violence in GB (Table-IV).

**Table-IV:** Risk factors of domestic violence.

Theme	f	%
Poverty	127	89
Influence of in-laws	95	67
Second marriage	71	50
Stepchildren	60	42
Forceful intimate relationships	35	25
Irresponsibility of husband	21	15
Husband drug addiction	21	15
Handicapped children	10	7
The demand for financial assistance from the wife’s family	8	6
The husband does not want children from me	8	6

## DISCUSSION

The current study shows higher prevalence of domestic violence in GB (88.8%). Among different types of abuse, psychological (69.4%) one was found to be highly prevalent among the study sample followed by physical (37.5%) and sexual violence (21.2%). These results are in accordance with the findings of other studies conducted in Pakistan but inconsistent with those reported from other contries.^6^,^12^,^15^ Another research conducted in Azad Kashmir, Pakistan that is more similar to GB from sociocultural perspective revealed that the psychological violence was found to be higher among women than physical violence.^16^

Finding that women who experienced domestic violence reported lower levels of mental health, psychological wellbeing, general positive affect, and life satisfaction and higher levels of psychological distress, anxiety, depression, and loss of emotional, behavioral control. Researchers from different socio-cultural and religio-political milieus reported similar findings i.e. domestic violence against women leads to their poorer psychological and emotional wellbeing, overall wellbeing, poorer mental health including depression, PTSD, anxiety, suicidality, alcohol abuse, drug abuse, eating disorders, psychosis, psychological distress, and psychological illness.^1^,^8^,^10^,^17^-^19^ Similar findings are reported by researchers from Pakistani context where different types of domestic violence; physical, psychological, and sexual strongly associated with higher levels of negative states of mental health including depression, mental tension, and hysteria.^5^,^20^

In the present study, the risk factors behind domestic violence were found as; poverty, the influence of in-laws, second marriage, stepchildren, forceful intimate relationships, husband’s irresponsibility, and addiction, handicapped children, husband’s demand of financial assistance from wife’s family, and husband’s reluctance for children. Similar findings were reported by Hossain from Bangladesh, i.e. more similar with Pakistan from religio-cultural perspectives, where role of in-laws and other family members, polygamous husbands, husbands’ addiction, husbands’ demand for dowry from in-laws, husbands’ cognitions that wives are product for their sexual enjoyment (nonconsensual sexual union), and giving birth to the female child were risk factors of domestic violence against women.^21^ Within Pakistani context, Bibi, Ashfaq, Shaikh, and Qureshi reported in-laws as perpetrators (30%) along with husbands’ drug addiction and extra marital relationship as risk factors.^11^ According to Shaikh, Shaikh, Kamal, and Masood, 21.1% women in Pakistan reported that their husband forced them to have nonconsensual sexual relationship supports our findings, i.e. forceful intimate relationships.^22^ Lois, David, and Zachary reported unwanted pregnancy by perpetrator along with lower socio-economic status as a risk factor of abuse is in line with our findings, i.e., the husband did not want children.^23^ However, we found poverty as a dominant risk factor of domestic violence in the present study, also endorsed by other researchers. For example, lower socioeconomic status was found as an important risk factor of domestic violence against women in Pakistan and elsewhere.^6^,^7^,^24^ Ali and Gavino grouped all risk factors of domestic violence in Pakistan into two major categories: intrinsic factors (income, substance abuse etc.) and extrinsic factors (socio-economic, political, & cultural system of Pakistan along with the influence of neighboring countries).^25^

### Limitations of the study

The present study documented only the wives’ perspective. Secondly, the sample was small and represented only a single province of Pakistan (GB).

## CONCLUSIONS

The prevalence of domestic violence in GB was at an alarming level, and it has significant impacts on women’s mental health. Poverty, the influence of in-laws, and second marriage were found as dominant risk factors behind domestic violence. To address the alarming level of domestic violence promulgation of legislation and community-based awareness programs against domestic violence and the establishment of rehabilitation centers with mental health services for the victims of domestic violence are strongly suggested. Despite vital contributions, the present study is not free from limitations like other scientific studies.

### Author`s Contribution

**HH & SH** conceived, designed and did data collection and statistical analysis & editing of manuscript. Both authors are responsible and accountable for the accuracy and integrity of the work.

**SZ & TH** did manuscript writing, review, and final approval of manuscript.
